# Risk assessment for job burnout with a mobile health web application using questionnaire data: a proof of concept study

**DOI:** 10.1186/s13030-016-0082-4

**Published:** 2016-11-02

**Authors:** Roland von Känel, Marc van Nuffel, Walther J. Fuchs

**Affiliations:** 1 Department of Psychosomatic Medicine, Clinic Barmelweid, CH-5017 Barmelweid, Switzerland; 2 Department of Neurology, Inselspital, Bern University Hospital, and University of Bern, Bern, Switzerland; 3 Neuropsychosomatic and Stress Biology Research Group, Department of Clinical Research, University of Bern, Bern, Switzerland; 4 Burnout Protector GmbH, c/o Barmelweidgruppe AG, Barmelweid, Switzerland; 5 DU DA Group GmbH, Development & Consulting, Zürich, Switzerland; 6 Digiboo® GmbH, Zürich, Switzerland

**Keywords:** Burnout, Depression, Fatigue, Mobile health, Psychological stress, Sleep, Work stress

## Abstract

**Background:**

Job burnout has become a rampant epidemic in working societies, causing high productivity loss and healthcare costs. An easy accessible tool to detect clinically relevant risk may bear the potential to timely avert the dire sequelae of burnout. As a start, we performed a proof of concept study to test the utilization of a mobile health web application for a free and anonymous burnout risk assessment with established questionnaires.

**Methods:**

We designed a client-side javascript web application for users who filled out demographic and psychometric data forms over the internet. Users were recruited through social media, back links from hospital websites, and search engine optimization. Similar to population-based studies, we used the Maslach Burnout Inventory-General Survey (MBI-GS) to calculate a burnout risk index (BRIX). As additional mental health burden indices, users filled out the Perceived Stress Scale, Insomina Severity Index, and Profile of Mood States.

**Results:**

Within six months, the MBI-GS was completed by 11,311 users (median age 33 years, 85 % women) of whom 20.0 % had no clinically relevant burnout risk, 54.7 % had mild-to-moderate risk, and 25.3 % had high risk. In the 2947 users completing all questionnaires, female sex (*B* = -0.03), cohabiting (*B* = -0.03), negative affect (*B* = 0.46), positive affect (*B* = -0.20), perceived stress (*B* = 0.18), and insomnia symptoms (*B* = 0.04) explained 56.2 % of the variance in the continuously scaled BRIX. The reliability was good to excellent for all psychometric scales. The weighting of the BRIX with mental health burden indices primarily modified the risk in users with mild-to-moderate burnout risk.

**Conclusions:**

A low-threshold web application can reliably assess the risk of job burnout. As the bulk of users had clinically relevant burnout scores, a web application may be useful to target employees at risk. The clinical value of the BRIX and its modification with coexistent/absent mental health burden awaits evaluation with work and health outcomes.

## Background

Job burnout due to chronic stress at the workplace puts a serious health and economic burden on individuals and societies. For instance, about half of European workers report stress to be common in their workplace, whereby stress contributes to around half of all lost working days [1]. In Switzerland, one out of four employees reported a significant amount of exhaustion, the core symptom of job burnout in 2010; already in 2000, work stress-related costs added up to CHF 4.2 billion, equaling the amount for the country’s military spending [2]. Similarly, 26 % of workers in the U.S. reported that they are often or very often burned out or stressed by their work [3], with workplace stress being responsible for up to $190 billion in annual healthcare costs in the United States [4].

Most often used definitions of job burnout rely on the Maslach Burnout Inventory (MBI) of which several versions exist [5]. The 16 items of the MBI-General Survey (MBI-GS) are formulated “occupation-neutral” which, due to its universal applicability to different professions, makes the MBI-GS the preferred version to measure symptoms of job burnout [6, 7]. The MBI-GS clusters in three scales [8]. The exhaustion scale describes feelings of emotional and physical resource depletion, while the cynicism scale refers to impersonal and distant attitudes towards one’s job. The professional efficacy scale describes subjective feelings of (low) achievements in one’s work. The three-factor structure of the MBI-GS has been confirmed among different occupational domains (e.g. health care, academic, or manufacturing), both cross-sectionally and longitudinally, and to be invariant across occupations and nations [9, 10]. There is a sense among experts that although total MBI scores are an indication for total symptom burden, the three subscales should also be investigated and interpreted as distinct constructs, although exhaustion designates the core component of the burnout syndrome [11]. The MBI with its three dimensions of the burnout experience that emerged from earlier qualitative research, has been considered the standard tool for research in the field, although it should be noted that different conceptualizations of burnout exist, with some measures focusing on, for instance, the exhaustion component alone [6].

In addition to job-related stress, demographic and psychological factors, including young age, low socioeconomic status, low social support, stress outside the workplace (i.e., at home or with financial issues), sleep problems, low positive affect (PA), high negative affect (NA), have also been associated with increased levels of burnout [11–16]. Women seem to score higher in exhaustion, whereas men usually show higher cynicism [11]. Some authorities, including the German Association for Psychiatry, Psychotherapy and Psychosomatics, feel burnout is not a disease on its own, but a “risk state” for ensuing somatic and psychiatric diseases, including cardiovascular diseases, functional somatic symptoms, depression, anxiety and substance use disorders [17].

Regardless this debate, a lack of considering important variables that clearly augment or attenuate burnout symptoms, may only insufficiently capture a dynamic perspective of burnout risk [18]. Specifically, as a novelty of the present study, we are not aware of any studies having tried to weight burnout risk by mental health factors that may explain substantial variance, including perceived global stress, subjective sleep quality and mood. For the prevention of burnout and its psychiatric and somatic consequences, the modification of current burnout risk by indices of mental health burden, which, by themselves, are modifiable through behavioral change (e.g. bettering sleep hygiene), might help to predict an individual employee’s chance to develop (or remit from) clinically relevant burnout symptomatology even with greater accuracy.

In the realm of electronic healthcare (eHealth) practices supported through the internet, uncomplicated access to a web-based guided self-help intervention to alleviate burnout symptoms is a timely means to potentially improve employee health, prevent sick leave, and lower health expenditures for companies and society.

Mobile health web applications are increasingly developed to assess and monitor health burden of users in a self-responsible manner [19]. The potential of such web applications to maintain or improve health, and best concepts to change health behaviors of users have only begun to being scrutinized, as mobile health has just started to change the way healthcare is delivered [20]. The possibility for an anonymous burnout assessment may be attractive if, for instance, users must fear disadvantages when communicating health problems to company medical officers.

We evaluated the use of a free web application for the anonymous assessment of burnout risk with established psychometric scales, including the MBI-GS. Our first aim was to show that a web application attracts and identifies individuals at risk for burnout. This is an important requirement so that those in need can ever initiate internet-guided interventions as preventive steps. Accordingly, our specific hypothesis was that an elevated burnout risk in users of the web application is more prevalent than would be expected in the general population, where the prevalence of mild-to-moderate and severe burnout is 25 % and 3 %, respectively [21]. Our second aim was to show that indices of mental health burden, namely perceived stress, insomnia and mood, explains a substantial variance in burnout scores, after controlling for demographic factors. While such a finding concurs with previous studies [13–16], the novelty is to show that collection of questionnaire data over the internet yields comparable results and, therefore, can usefully be employed in the context of eHealth.

As a third aim, we explored the extent to which the weighting of burnout risk by indices of the concomitant mental health burden (i.e., perceived stress, insomnia, and mood) modified users’ burnout risk to evaluate the validity of such an approach for burnout prevention and better work- and health-related outcomes, which hypotheses had to be tested in future prospective studies.

## Methods

### Study participants

The participants of this study were 11,311 consecutive subjects (in the following termed “users”) who between 05/27/15 and 11/30/15 utilized a mobile web application to anonymously assess and monitor their risk of job burnout with the MBI-GS over the internet. Here, we report on cross-sectional data, because, within the 6-month survey, only 41 users completed the MBI-GS twice and three completed it four times. The web application comes as a free online tool, termed “Burnout Protector™” (cf. online link under [22]) and is available in English and German. This brand name points out the web application’s ultimate goal, that is to protect employees of different professions from burning out through early identification of a burnout risk state and initiation of appropriate means.

Exclusion criteria were age below 18 or above 99 years and objection to provide demographic data in terms of sex, cohabitation, and educational level. Occupational state, employment grade, psychiatric and medical history, and previous treatments for psychological problems were not exclusion criteria to increase external validity of our study findings. That is, we sought to obtain results from a heterogeneous population that can be generalized to users responding to this web application for burnout risk assessment at large.

With the premise of a low budget (CHF 4200), we recruited users through various means, including a) social media entrances (Facebook and Twitter); with, based on the performance of these entrances, b) subsequent Facebook adds, targeting women who had expressed an interest in or liked pages related to “Education”, “Primary school”, “Health System”, “Hospital”, “Medicine”, “Physician”, “Public Health”, and “Nursing”; c) backlinks from partner institutions (i.e., rehabilitation clinics) presenting a Burnout Protector™ icon on their websites); and d) Google search engine optimization to improve visibility of the web application.

The data was surveyed and evaluated in accordance with scientific standards specified by Switzerland’s Federal Act on Research Involving Human Beings [23] and in compliance with the national ethical guidelines on the handling of personal data in the field of medicine issued by the Federal Data Protection Commissioner [24]. Accordingly, all users were informed upfront on the website that their anonymously provided health-related personal data could be used in coded form for research purposes. They were also informed that they could delete their data anytime with a single mouse-click if they did not consent with the anonymous data collection for research purposes. The following text was provided on the website: *“The test is free of charge. In return, we may use your anonymized data for research purposes. Only the user of the burnout test knows the true identity behind his or her personal data. The anonymous data is surveyed and evaluated in accordance with Swiss scientific standards as specified by the Federal Act on Research Involving Human Beings”* with a link to that website [23], where it says (text provided online): *“Further use may be made of non-genetic health-related personal data in coded form for research purposes if the person concerned or the legal representative or next of kin have been informed in advance and have not dissented.”* Importantly, such anonymously collected non-genetic health-related personal data do not require approval by an ethical committee in Switzerland. After subjects had completed online questionnaires and had obtained an estimate of their burnout risk, they were shown the question *“Delete all data?”* together with the icon *“Yes, please delete all my data”*. If they clicked the latter, the sentence *“Your data has been deleted”* popped up on the screen. Deleted data were not used for any analysis.

### Data collection with the web application

**Fig. 1:**
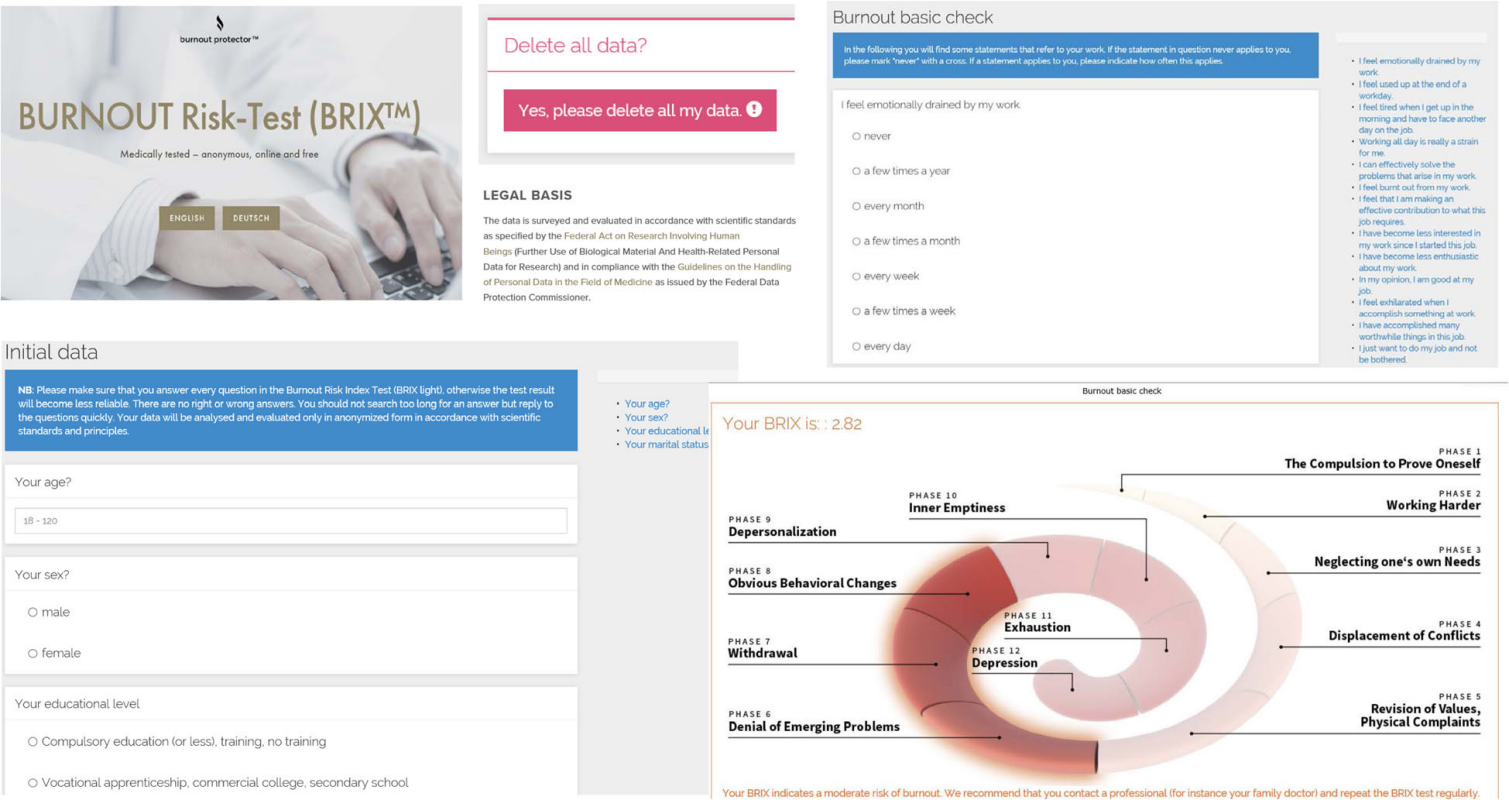
Screen shot samples of the web application. Legend: Depicted are screen shots of the login page, online forms for data collection, graphical feedback on the burnout risk index (BRIX score of 2.82 in this case) with recommendations, and about the legal basis and consent process

**Fig. 2:**
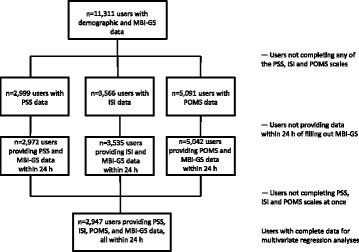
Flowchart of users of the web application. Legend: ISI, Insomnia Severity Index; MBI-GS, Maslach Burnout Inventory-General Survey; POMS, Profile of Mood States; PSS, Perceived Stress Scale

After login to the web application over the internet (cf. [22] for weblink), users who participated in this study were asked to fill one online form with demographic data and four online forms with psychometric data (burnout symptoms, perceived stress, insomnia symptoms, and mood) in either English or German. Figure 1 shows screen shot samples of the web application. To be delivered an estimate of burnout risk, at least forms for demographic factors and the MBI-GS had to be completed at the first login. At any time later, users could login and complete one or more forms according to their own judgment. However, users had to fully complete a form before the data could be saved and stored in the data repository for further analysis. In other words, there were no missing items once a form was completed. The web application can be completed repeatedly and users were prompted to do so if burnout risk was increased, so that they can track changes in their burnout risk. The web application is a modern, client-side, javascript application. It is built with Node.js, but is statically served. Due to the sensitive nature of the user data, the actual data is only stored locally within the browser, but each survey is submitted anonymously to a Splunk instance. Figure 2 shows the flow chart of users in the study with the sample sizes being available for the different analyses.

### Demographic factors

The users of the web application filled in indications about age (range 18-99 years), sex, cohabitation (living together with someone: yes/no), and highest level of education: I) compulsory education or less, training, no training; II) vocational apprenticeship, commercial college, secondary school; III: school leaving exam, vocational school certificate; and IV: university of applied sciences, technical college, university degree.

### Psychometric scales

#### Maslach burnout inventory

We used the MBI-GS that has been developed to estimate burnout risk in various professions as a crisis in employees’ relationship with work, but not necessarily with people at work [8]. The MBI-GS comprises 16 items of which five each refer to exhaustion and cynicism, and six to professional efficacy. The three-dimensional concept of the MBI-GS has been confirmed across nations and occupational groups [10] and validated when response items are filled in over the internet [25]. Typical items are “I feel emotionally drained by my work” for exhaustion, “I have become less enthusiastic about my work” for cynicism, and “In my opinion, I am good at my job” for personal efficacy. Higher scores on the exhaustion and cynicism subscales and lower scores on the professional efficacy subscale are indicative of burnout, whereby items for professional efficacy are reversed (low professional efficacy). Each item is rated on a 7-point Likert scale from 0 (never) to 6 (daily). Item scores were added up to obtain MBI-GS total scores and scores for each subscale. Several German translations of the MBI-GS exist, although, to our knowledge, no version has officially been validated. We consulted with previous German translations [26, 27] to build a MBI-GS German version (to be validated elsewhere). In the 11,311 users, Cronbach’s alpha was 0.88 for the MBI-GS total score, 0.88 for exhaustion, 0.84 for cynicism and 0.79 for professional efficacy, indicating good reliability.

#### Perceived stress scale

We used the 10-item Perceived Stress Scale (PSS), applying versions in English [28] and German [29]. The PSS has been designed as a global measure of appraised stress in the previous month. Although not specifically asking about the job environment, queries about feelings of being overwhelmed and losing control consider important aspects of the job-demand-control model according to which work stress results from an imbalance between high job demands and low decision latitude. This is alluded to with items such as “How often in the past month did you feel you could not cope with what you had to accomplish?” reflecting high demands, and “How often in the past month did you feel you could not control important things in life?” reflecting low control. Each item is rated on a 5-point Likert scale from 0 (never) to 4 (very often). Total scores of 0-13, 14-19, and 20-40, respectively, indicate low, medium, and high levels of perceived stress, and were assigned weighting factors of 1.0, 1.2, and 1.4 to modify burnout risk. In the 2999 users who completed the PSS, Cronbach’s alpha was 0.91, indicating excellent reliability.

#### Insomnia severity index

We used the 7-item Insomnia Severity Index (ISI) to rate difficulties falling and staying asleep, problems waking up to early, satisfaction with and worries about one’s sleep, and interference of sleep problems with daily functioning and quality of life, applying versions in English [30] and German [31]. Each item is rated on a 5-point Likert scale from 0 (no problem) to 4 (very severe problem). Total scores of 0-7, 8-14, and 15-28, respectively, indicate absence of clinically significant insomnia, subthreshold insomnia, and moderate-to-severe clinical insomnia, and were assigned weighting factors of 1.0, 1.3, and 1.6 to modify burnout risk. In the 3566 users who completed the ISI, Cronbach’s alpha was 0.87, indicating good reliability.

#### Profile of mood states

We applied the 35-item Profile of Mood States (POMS) questionnaire to describe current mood, whereby covering dimension of both positive affect (PA: vigor) and negative affect (NA: depression, fatigue, hostility), applying versions in English [32] and German [33]. Each item is rated on a 5-point Likert scale from 0 (not at all) to 4 (extremely). In the 5091 users who completed the POMS, Cronbach’s alpha was 0.90 for the PA scale (7 items for vigor), 0.97 for the NA scale (28 items for depressive mood, hostility and fatigue altogether), 0.95 for depressive mood (14 items), 0.92 for hostility (7 items), and 0.93 for fatigue (7 items), indicating excellent reliability. A ratio of perceived PA-to-NA of about 3:1 has been proposed as an index of flourishing mental health [34]; therefore, we assigned weighting factors of 0.8, 1.0, 1.2, 1.4, and 1.6 to PA/NA ratios of ≥3.0; ≥2.0 but <3.0; ≥1.0 but <2.0; ≥0.5 but <1; and <0.5, respectively, to modify burnout risk. We use the following formula to compute the PA/NA ratio: [3.0 x PA score / (0.5 x depression score + 1.0 x hostility score + 1.0 x fatigue score)]. The 20 users scoring “0” for NA were assigned a score of “1” to enable calculation of the PA/NA ratio.

### Burnout risk index

#### Burnout risk index-original (BRIX-O)

With reference to previous population-based studies [21, 35], we applied the following formula to compute burnout syndrome scores: [(0.40 x exhaustion) + (0.30 x cynicism) + (0.30 x low professional efficacy)]. With this method, burnout syndrome scores correspond to the original response scale (0-6) and weight exhaustion as the primary symptom dimension. Based on these burnout syndrome scores, termed Burnout Risk Index-Original (BRIX-O) for the purpose of the web application, we categorized users into three groups with differing BRIX-O scores: I) no clinically relevant burnout risk (0-1.49); II: mild-to-moderate burnout risk (1.50-3.49); III: high burnout risk (3.50-6) [21, 35]. These cutoffs are of clinical relevance because population-based studies showed significantly reduced mental health (e.g., higher prevalence of major depression) and physical health (e.g. higher prevalence of musculoskeletal and cardiovascular illnesses) in individuals with burnout scores of 1.50 or higher compared to those with scores below 1.50 [21, 36].

#### Burnout risk index-weighted (BRIX-W)

If users completed the PSS, ISI, and POMS, we assigned weighting factors based on the severity of perceived stress, insomnia symptoms, and mood disturbance as specified above. These psychometric measures were selected and weighting factors were defined on the knowledge base that perceived stress (both at work and outside work), poor subjective sleep and mood disturbances are indices of mental health burden that are reliably associated with burnout [13–16]. As studies suggest greater predictive value of insufficient sleep than mood (e.g. depression) for the development of clinical burnout [37], we assigned insomnia symptoms relatively greater weight than mood disturbances. The multiplication of the BRIX-O score with either one, two or three weighting factors yielded a modified or weighted burnout risk index (BRIX-W). The maximum upper limit of the BRIX-W score was 21.5 based on the formula [6 (for the maximum BRIX-O score) x 1.4 (for high level of perceived stress) x 1.6 (for high level of insomnia symptoms) x 1.6 (for high level of mood disturbance)]. The value of the BRIX-W has not been tested empirically. We assumed the cut-offs to define the level of burnout risk with the BRIX-W to be identical to the BRIX-O. That is, a given score of a burnout measure will increase with each additional contributing factor of mental health burden that is present at a critically high level. In contrast, “stress-buffering” properties of PA should lower burnout scores.

### Statistical analyses

Data were analyzed using SPSS 23.0 statistical software package (SPSS Inc. Chicago, IL) with level of significance at *p* < .05 (2-tailed). Differences between groups were calculated using one-way analysis of variance, Kruskal-Wallis test or Pearson chi-square test for continuously and categorically scaled variables, respectively. Independent-samples t-test and Mann-Whitney U test were applied for post-hoc group comparisons. Pearson correlation analysis was used to estimate the relationships between two variables. Linear regression analyses, using forced entry of dependent variables, were employed to identify which demographic variables and psychometric scales were significantly linked with burnout measures, with the BRIX-O as the primary outcome. A variance inflation factor (VIF) above 2.5. was considered to indicate critical multicollinearity between dependent variables.

## Results

### Characteristics of web application users

Table 1 summarizes the demographic and psychometric characteristics of the 11,311 users of the web application. The sample was predominantly female with a median age of 33 years (range 18-99). The majority of users lived in a partnership and was well educated. Based on the BRIX-O score, 20.0 % did not have a clinically relevant burnout risk (Group I: score 0-1.49), whereas 54.7 % had a mild-to-moderate risk (Group II: score 1.50-3.49) and 25.3 % had a high risk (Group III: score 3.50-6). Accordingly, mental health burden was high in the sample. Specifically, of the 2999 and 3566 users who also completed the PSS and the ISI, respectively, six out of seven reported elevated levels of perceived stress (PSS score ≥14) and two out of three had clinically relevant insomnia symptoms (ISI score ≥8). Likewise, three out of five users providing POMS data, did not reach a PA/NA ratio of at least 1.

**Table 1:** Demographic and psychometric characteristics of all 11,311 users of the web application

Variables	All users	Users per BRIX-O group (score, range)			*P*
	*n* = 11,311	I (0-1.49) *n* = 2259	II (1.50-3.49) *n* = 6188	III (3.50-6.00) *n* = 2864	
*Demographics (n = 11,311)*					
Age (yrs), mean (SD)	34.76 (10.38)	36.77 (10.94)	34.02 (10.08)	34.77 (10.36)	<.001
Female sex, n (%)	9597 (84.8)	1817 (80.4)	5339 (86.3)	2441 (85.2)	<.001
Cohabiting (yes), n (%)	8417 (74.4)	1253 (78.0)	4595 (74.3)	2076 (72.5)	<.001
Educational level, n (%)^a^					<.001
I	1372 (12.1)	175 (7.7)	719 (11.6)	477 (16.7)	
II	5526 (48.9)	1097 (48.6)	3030 (49.0)	1399 (48.8)	
III	1652 (14.6)	273 (12.1)	942 (15.2)	437 (15.3)	
IV	2762 (24.4)	714 (31.6)	1497 (24.2)	551 (19.2)	
*MBI-GS scores (n = 11,311)*					
MBI total, mean (SD)	39.66 (18.18)	14.76 (5.43)	37.93 (8.80)	63.04 (8.48)	<.001
BRIX-O, mean (SD)	2.62 (1.18)	0.98 (0.34)	2.51 (0.57)	4.14 (0.49)	<.001
Exhaustion, mean (SD)	16.88 (7.76)	6.88 (3.42)	16.60 (5.49)	25.37 (3.55)	<.001
Cynicism, mean (SD)	12.72 (8.15)	3.74 (2.92)	11.33 (5.42)	22.81 (4.72)	<.001
Low PE, mean (SD)	10.06 (7.20)	4.13 (3.87)	10.00 (6.42)	14.86 (7.28)	<.001
*PSS scores (n = 2999)*					
PSS total, mean (SD)	22.87 (7.92)	14.64 (7.39)	22.91 (6.45)	28.54 (5.31)	<.001
PSS categories, n (%)					
Scores 0-13	442 (14.7	288 (49.1)	143 (9.1)	11 (1.3)	
Scores 14-19	461 (15.4)	144 (24.5)	287 (18.3)	30 (3.6)	
Scores 20-40	2096 (69.9)	155 (26.4)	1140 (72.6)	801 (95.1)	
*ISI scores (n = 3566)*					
ISI total, mean (SD)	11.46 (6.33)	6.94 (5.19)	11.27 (5.85)	15.11 (5.74)	<.001
ISI categories, n (%)					
Scores 0-7	1080 (30.3)	439 (61.9)	540 (28.6)	101 (10.4)	
Scores 8-14	1292 (36.2)	205 (28.9)	748 (39.7)	338 (34.8)	
Scores 15-28	1194 (33.5)	65 (9.1)	597 (31.7)	532 (54.8)	
*POMS scores (n = 5091)*					
Vigor, mean (SD)	8.35 (5.70)	13.11 (6.06)	8.11 (5.09)	5.28 (3.96)	<.001
Depression, mean (SD)	16.82 (12.77)	6.19 (7.46)	15.04 (10.62)	27.47 (11.82)	<.001
Hostility, mean (SD)	8.65 (6.62)	3.83 (4.36)	8.23 (5.83)	13.08 (6.65)	<.001
Fatigue, mean (SD)	14.12 (7.22)	7.22 (5.16)	13.93 (6.21)	19.63 (5.65)	<.001
Positive affect, mean (SD)	25.06 (17.09)	39.33 (18.18)	24.33 (15.26)	15.84 (11.88)	<.001
Negative affect, mean (SD)	31.18 (18.07)	14.15 (11.42)	29.86 (14.74)	46.44 (15.27)	<.001
PA/NA ratio, median (IQR)	0.70 (0.32, 1.83)	3.43 (1.59, 7.00)	0.75 (0.38, 1.50)	0.33 (0.14, 0.54)	<.001
PA/NA ratio categories, n (%)					
Ratio ≥3.0	859 (16.9)	584 (56.8)	267 (10.0)	8 (0.6)	
Ratio ≥2.0 but <3.0	334 (6.6)	127 (12.3)	202 (7.5)	5 (0.4)	
Ratio ≥1.0 but <2.0	811 (15.9)	163 (15.9)	577 (21.5)	71 (5.1)	
Ratio ≥0.5 but <1.0	1105 (21.7)	78 (7.6)	724 (27.0)	303 (22.0)	
Ratio <0.5	1982 (38.9)	76 (7.4)	913 (34.0)	993 (71.9)	

*BRIX-O* burnout risk index-original, *IQR* interquartile range, *ISI* Insomnia Severity Index, *MBI-GS* Maslach Burnout Inventory-General Survey, *NA* negative affect, *PA* positive affect, *PE* professional efficacy, *POMS* Profile of Mood States, *PSS* Perceived Stress Scale, *SD* standard deviation

^a^Educational levels refer to compulsory education (or less), training, no training (level I); vocational apprenticeship, commercial college, secondary school (level II); school leaving examination, vocational school certificate (level III); and university of applied sciences, technical college, university degree (level IV)

*P*-values refer to comparisons between the three BRIX-O categories. Post-hoc comparisons between individual BRIX categories on all continuously scaled variables were significant at *p* < .001

Demographic and psychometric comparisons between the three BRIX-O categories revealed higher percentage of men and of cohabiting and higher educated users in the group without clinically relevant burnout risk. The users of that group were also somewhat older. Expectedly, exhaustion, cynicism, and low professional efficacy increased significantly across the three BRIX-O groups, with highest scores seen in the group with a high burnout risk. Similar significant group differences were observed for perceived stress, insomnia severity, NA as a whole, but also for depression, hostility, and fatigue scores individually. In turn, PA (i.e., vigor) and the PA/NA ratio decreased from the group with no burnout risk to the group with a mild-to-moderate risk and the one with a high burnout risk.

### Correlations with burnout symptoms

For the subsequent analyses, we excluded 27, 31, and 49 users, respectively, who did not fill out the PSS, ISI, and/or POMS forms within 24 h of completing the MBI-GS, because relevant changes, for instance in mood, might occur within this time interval. The results for the continuously scaled BRIX-O score concurred with those using BRIX-O categories. Higher levels of continuously scaled burnout symptoms were associated with higher levels of perceived distress, insomnia symptoms, and NA (r-values between 0.23 and 0.69) and with lower PA and PA/NA ratio (r-values between -0.35 and -0.66). There also were direct associations among perceived stress, insomnia symptoms and NA (r-values between 0.50 and 0.74), while higher levels of perceived stress, insomnia symptoms and NA were associated with lower PA and PA/NA ratio (r-values between -0.35 and -0.76). All of the above correlations were significant at *p* < 0.001.

An inspection of the size of the correlation coefficients in users who had completed two respective questionnaires at a time and among the 2947 users of the web application who had completed all questionnaires (i.e., MBI-GS, PSS, ISI, and POMS) showed negligible differences (all p-values <0.001). Therefore, we may assume that the results from the subsequent linear regression models, calculated from the sample of these 2947 users, might have looked similar in the full sample of 11,311 users, had all of these provided complete psychometric data.

### Multivariate associations with burnout symptoms

In the entire sample of the 11,311 web application users demographic factors explained little of the variance (between 0.9 and 2.0 %) in burnout symptoms (data not shown in detail). Similar effects were observed for demographic factors in the 2947 users with complete data (Table 2, Model 1).

**Table 2:** Demographic and psychometric factors regressed on burnout symptom scores (*n* = 2947)

Variables entered	Burnout Risk Index-Original		Exhaustion		Cynicism		Low professional efficacy	
	Model 1	Model 2	Model 1	Model 2	Model 1	Model 2	Model 1	Model 2
	B	B	B	B	B	B	B	B
Age (yrs)	-.045*	-.011	-.044*	-.011	.000	.030*	-.075***	-.060**
Female sex	.018	-.029*	.041*	-.005	-.014	-.048**	.009	-.026
Cohabiting	-.073***	-.026*	-.052**	-.004	-.076***	-.036*	-.052**	-.030
Education	-.125***	.010	-.121***	.013	-.090***	.019	-.087***	-.017
PSS (score)		.177***		.200***		.101***		.111***
ISI (score)		.040*		.089***		.016		-.042
NA (score)		.459***		.400***		.462***		.223***
PA (score)		-.203***		-.173***		-.137***		-.196***
Statistics for Model 2	Adjusted *R* ^*2*^ = .562		Adjusted *R* ^*2*^ = .529		Adjusted *R* ^*2*^ = .389		Adjusted *R* ^*2*^ = .187	
	F_8,2938_ = 473.66		F_8,2938_ = 414.37		F_8,2938_ = 235.93		F_8,2938_ = 85.65	
	*P* < .001		*P* < .001		*P* < .001		*P* < .001	

The columns show standardized coefficients Beta (B) with significance level: ***p < .001, **p < .010, *p < .050; using forced entry of demographic factors in Model 1 and additionally of psychometric scores in Model 2. For educational level, four categories were entered (4 = highest level of education; 1 = lowest level of education)

*ISI* Insomnia Severity Index, *NA* negative affect, *PA* positive affect (i.e., vigor), *PSS*, Perceived Stress Scale

However, these relationships notably changed with the additional consideration of perceived stress, insomnia symptoms and mood in Model 2 (Table 2). Psychometric scales accounted much for the elevated level of burnout symptoms in users with lower educational level and, to a lesser extent, in those living alone. The relations of age and sex with burnout symptoms were also somewhat modified by psychometric scales. The fully adjusted Model 2 suggested that the total level of burnout symptoms (BRIX-O score) was higher in men than women. DP was increased in men, but also in older users, while younger users scored higher in LA.

The variables in Model 2 together explained more than half of the variance in the BRIX-O and exhaustion; less variance was explained for cynicism and professional efficacy. High levels of NA contributed most to the variance in the BRIX-O and all MBI-GS subscales. Further significant contributions were made by high levels of perceived stress and low levels of PA. Insomnia symptoms significantly contributed to the BRIX-O and exhaustion, but not cynicism and professional efficacy.

As an additional and last step, we performed an exploratory analysis, whereby adding an interaction term between sex and PSS, ISI, NA and PA scores to Model 2, performing four separate analyses. None of the interactions turned out to be significant (all *p*-values ≥ .10), suggesting that men and women did not significantly differ in the observed associations of perceived stress, insomnia symptoms, and mood with the BRIX-O and MBI-GS subscales.

### Supplementary multivariate analyses

#### Addressing multicollinearity

Because there was multicollinearity for NA and perceived stress (VIF for PSS: 2.72, VIF for NA: 2.55), we rerun Model 2 (Table 2) with either scale alone. *Dropping the PSS score* did not substantially change the amount of variance in burnout measures explained by all variables in the model; whereby NA compensated for somewhat more of the variance (BRIX-O: *B* = 0.552, exhaustion: *B* = 0.505, cynicism: *B* = 0.515, low professional efficacy: *B* = 0.281) that was previously explained by PSS, than did PA and insomnia symptoms. In turn, *dropping the NA scale* substantially increased the individual variances now explained by perceived stress (BRIX-O: *B* = 0.434, exhaustion: *B* = 0.424, cynicism: *B* = 0.360, low professional efficacy: *B* = 0.235) and insomnia symptoms regarding the BRIX-O (*B* = 0.171) and exhaustion (*B* = 0.203), with, again, minor reduction in the total explained variance of burnout symptoms. VIFs were <2.0 in both these supplementary models.

#### Replacing NA and PA scores by the PA/NA ratio

The total amount of variance explained in burnout measures was similar to the original fully adjusted model. However, compared to the combined variance explained by PA and NA (Table 2), the PA/NA ratio alone made a smaller contribution (BRIX-O: *B* = -0.407, exhaustion: *B* = -0.383, cynicism: *B* = -0.329, low professional efficacy: *B* = -0.257) which, in turn, was compensated by an increase in the variance explained by perceived stress and insomnia symptoms.

#### Substituting NA scores by depression, hostility or fatigue scores

The three dimensions of NA explained somewhat less of the individual variance when compared with the aggregated NA scale as presented in Table 2. The following significant coefficients emerged for depressive mood (BRIX-O: *B* = 0.386, exhaustion: *B* = 0.228, cynicism: *B* = 0.428, low professional efficacy: *B* = 0.321), hostility (BRIX-O: *B* = 0.277, exhaustion: *B* = 0.211, cynicism: *B* = 0.304, low professional efficacy: *B* = 0.152) and fatigue (BRIX-O: *B* = 0.341, exhaustion: *B* = 0.433, cynicism: *B* = 0.275). Eventually, a model with all four dimensions of the POMS entered together with demographic factors, perceived stress and insomnia symptoms was hampered by multicollinearity, and so was not pursued further.

### Weighting of the BRIX score by psychometric scales

The proportion of users who completed the MBI-GS only (*n* = 6199), one or two psychometric scales additionally (*n* = 2136), or all three scales additionally (*n* = 2976), differed significantly between burnout risk groups (*p* < .001). Particularly, the proportion of users who completed all three scales was higher in the high risk group (29.2 %) than in the mild-to-moderate risk group (25.2 %) and the group without burnout risk (25.8 %); this suggests that users with a BRIX-O in the range of a high burnout risk were more often prompted to fill in psychometric questionnaires in addition to the MBI-GS.

**Fig. 3:**
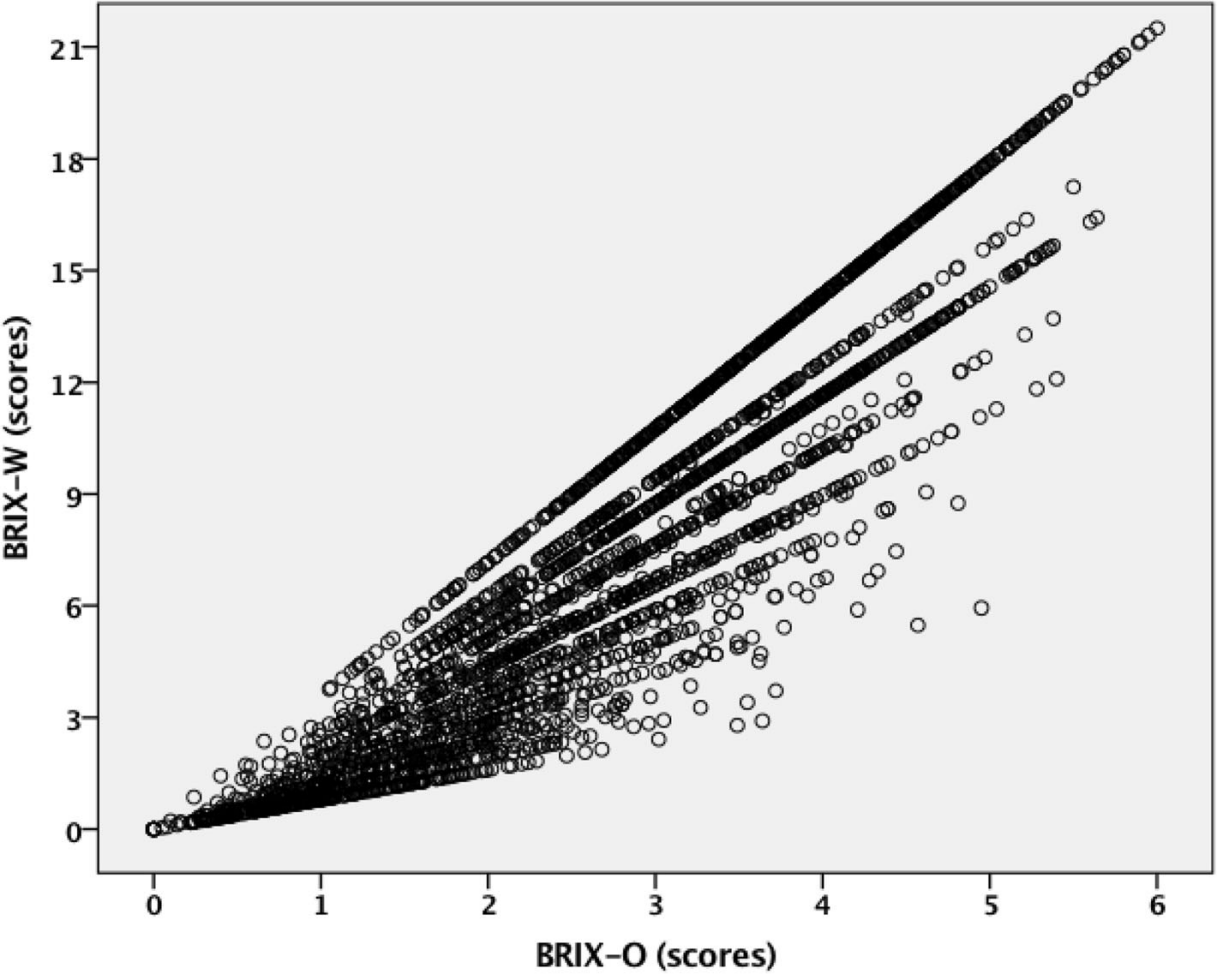
Association between original and weighted BRIX scores; Legend: The scatterplot shows the correlation between burnout risk index original (BRIX-O) scores calculated from exhaustion, cynicism and low professional efficacy scores, and burnout risk index weighted (BRIX-W) scores that are BRIX-O scores weighted with the severity of perceived stress, insomnia symptoms and mood disturbance (*r* = .93, *p* < .001)

Figure 3 shows the scatterplot for the BRIX-O (range 0-6) and the BRIX-W (range 0-21.50) in the 2947 users who provided complete psychometric data within the same 24 h-interval (*r* = .93, *p* < .001). Adjustment for age, sex, cohabiting, and educational level did not change the strength of this relationship (*r* = .93, *p* < .001).

**Fig. 4:**
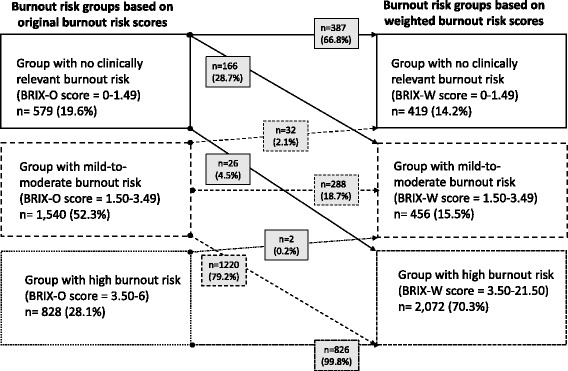
Changes in burnout risk after weighting with indices of mental health burden. Legend: Groups and numbers (n, %) of users who experienced a shift or no shift in their burnout risk after burnout risk index original (BRIX-O) scores were weighted (BRIX-W) with perceived stress, insomnia symptoms and mood disturbance

Figure 4 depicts the number and proportion of users who experienced a shift in their burnout risk with modification of the BRIX-O. In users without burnout risk, 28.7 % and 4.5 % were reclassified to have mild-to-moderate and high risk, respectively. Among those with mild-to-moderate risk, 79.2 % were reclassified to have high risk. Very few users experienced a reduction in their burnout risk, with 2.1 % shifting from the group with mild-to-moderate risk to the group with no risk, and with only 0.2 % of users with a high risk shifting to the group with a mild-to-moderate risk. There were no users experiencing a shift from the high to the no risk group. The weighting of the BRIX-O with the three indices of mental health burden thus primarily modified the risk in users with mild-to-moderate and, to a lesser extent, with no burnout risk, but not in those with a high risk.

## Discussion

We showed that a mobile health web application is useful to assess the risk of job burnout. For the sake of keeping user reactance possibly low, we designed the web application as a free and anonymous low-threshold tool with limited time expenditure needed for burnout risk assessment. This requirement precluded collection of an abundant amount of data, including detailed questions on the work situation, medical and psychiatric history, medication use and previous psychological interventions. At the same time, we were anxious to comply with up-to-date scientific and clinical knowledge in the field of job burnout that can universally be applied to male and female workers in different occupations, age groups and nations, guiding us to use the MBI and validated psychometric scales to measure perceived stress, sleep quality and mood.

Despite a low budget available for social media entrances and targeting persons with an assumed interest in our web application, we were able to attract 11,311 users during a surveyed period of six months. Compared to representative population-based samples, where about 30 % of workers report clinically relevant burnout, with up to 5 % having severe burnout [21, 38], we found this prevalence to be much higher in our non-representative sample. Applying previously suggested cut-offs to estimate burnout risk with the MBI-GS [21, 35], a staggering 80 % of users of our web application had clinically relevant burnout, of which 25 % reported symptom scores in the range of severe burnout. Hence, the prevalence of clinically relevant burnout risk in workers using our web application was at least 3-times higher than can normally be expected in a working population. In other words, workers at risk for clinically relevant job burnout could clearly be reached with this web application. Also, of all users, 26 % filled out all psychometric forms, with preponderance of those with high burnout risk; perhaps, because they were personally more affected by elevated BRIX-O score. Accordingly, mental health burden was high in the sample, with at least 90 % of users with a high burnout risk reporting clinically relevant levels of perceived stress, insomnia symptoms and mood disturbance. These observations are important regarding public health implications, because becoming aware of one’s burnout risk and mental health burden is a premise to initiate preventive and therapeutic steps. These include changing work and lifestyle behavior, talking to supervisors and seeking professional help, including primary care visits for a thorough assessment of numerous diseases with clinical presentation of disturbing fatigue [17, 39]. Currently, the web application simply recommends consultation with a professional (e.g. family doctor) if the assessment yields an increased risk for burnout. Where geographical boundaries exist, the web application could also be used for telemedicine purposes in that a doctor or mental health specialist provides instructions over the internet. As a next step, pursuing an eHealth approach, we will provide specific recommendations for interventions to alleviate burnout symptoms for users with a clinically relevant burnout risk over the internet.

The reliability of the applied psychometric scales was good to excellent, indicating their validity for the measurement of burnout risk, appraised global stress, insomnia symptoms and mood (disturbance) in our sample. There were expected significant and direct relationships among burnout, stress, insomnia, and NA scores, whereas inverse relationships were found with PA scores. The multivariate model was able to explain 56.2 % of the variance in the BRIX-O score, our primary outcome. Regarding subscales, most of the variance was explained for exhaustion, followed by cynicism and low professional efficacy. We emphasize that a set of three psychosocial scales, covering stress appraisal, sleep disturbance and mood, was sufficient to explain a substantial proportion of the variance in burnout risk. In contrast, demographic factors explained negligible variance. Low socioeconomic status and living alone, an index of low social support, were expectedly associated with greater scores in virtually all burnout measures. The association of mental health burden indices with burnout measures did not differ between sexes, so our web application might equally be applied to male and female workers. Nonetheless, because 85 % of the web application users were women, it is possible that significant differences between women and men would had emerged with a greater proportion of men among users. Moreover, male users scored higher in the BRIX-O and cynicism scores, but were equal in exhaustion scores, when compared with female users. This partially contradicts a previous meta-analysis which also found somewhat higher cynicism in men, but slightly higher exhaustion in women and no gender differences in overall measures of job burnout [40]. Therefore, it is possible that burnout risk was skewed towards higher values in the comparably small subgroup of male users. A greater proportion of male users with a lower burnout risk might have yielded different results for men and women regarding the associations between mental health burden indices and burnout measures. As the observed associations of demographics and psychometric scales with burnout measures are similar to those seen in non-web based studies, we may assume that the users of our web application provided accurate indications about their mental health state. In general, results from psychological assessments with online questionnaires are fairly equivalent to their paper-and-pencil versions [41].

Burnout scores have been shown to correlate with depressive mood in numerous studies [21, 42]. However, our aggregate measure of NA, comprising depressive mood, hostility and fatigue, explained somewhat more of the variance in BRIX-O scores than did depressive mood, fatigue and hostility individually. Considering a range of NA types thus may be more valuable to predict burnout risk (and vice versa) than assessment of depressive mood alone. PA scores, referring to vigor in our study, explained about half the variance of burnout scores when compared with NA. Moreover, PA emerged as an equally important predictor of BRIX-O scores and all MBI-GS subscales compared with perceived stress. This concurs with the mainstream of “positive psychology” research, suggesting PA is not the mere opposite of NA, but exerts a myriad of health benefits that are independent of NA [43, 44]. Insomnia symptoms explained comparable little variance in the BRIX-O score, whereby particularly contributing to exhaustion. It was suggested that NA accounted for a considerable amount of the relationship between insomnia symptoms and burnout scores.

BRIX-O scores and BRIX-W scores were highly correlated. This concurs with the observation that mental health burden drastically increased across the three groups with increasing burnout risk. The weighting of the BRIX-O score with the severity of perceived stress, insomnia symptoms, and imbalance between PA and NA, resulted in reclassification of workers mostly in the mild-to-moderate burnout risk group, whereby almost 80 % shifted to the high risk group. One third of users without clinically relevant burnout risk initially shifted to a higher risk category, with the vast majority ending up in the mild-to-moderate risk group. In contrast, very few users experienced a reduction in burnout risk. Likely due to the fact that the applied algorithm only allows the BRIX-O score to drop, if users endorse a PA-to-NA ratio of at least 3.0 that is associated with mental flourishing [34]. Therefore, we will consider additional protective measures in future studies, such as a high amount of regular physical activity [45] and a healthy stress response, indexed by measures of high autonomic nervous system and hypothalamic-pituitary adrenal axis functioning [46].

Currently, the BRIX-W can only identify users with a probable high risk to develop clinically relevant burnout symptomatology in the mid-to-longer-term due to coexistent high mental health burden. Whether the BRIX-W has greater clinical value than the BRIX-O awaits evaluation with work and health outcomes in prospective studies. However, regarding user reactance, this planned “upgrade” in data collection may be problematic, such that additional work and health assessments, including detailed exploration of the working situation, ought to be offered as complementary options only, even though this interferes with high requirements for cutting-edge research in the field. Nevertheless, the ultimate goal is to provide each user with a vulnerability index that integrates changes in stress, sleep and mood, based on which individual prediction can be made as to the development or remittance of burnout risk in a given time interval. Repeated data assessments and big data concepts may enable us to achieve this ambitious goal [47].

The large sample size, use of valid and widely used instruments applied in stress research, and lack of missing questionnaire items are notable strengths of our study, which, however, had also its limitations, inherent to the study design. This was a non-representative sample that comprised predominantly women and higher educated users who were largely recruited through electronic media. Therefore, our results should not be generalized to the working population at large and employees with low web literacy. Specifically, eHealth tools are only of value if users have the skills to effectively engage in them over the internet [48].

Avoiding user reactance comes at the cost of omitting correlates of burnout, such as personality characteristics, lifestyle factors, and specific measures of work stress, which all might explain additional variance in burnout scores. Also due to anticipated user reactance, we felt declined to perform clinical interviews that had increased the reliability of self-report psychological measures. Noteworthy multicollinearity between NA and PSS limits interpretation about the individual predictive power of each scale for the BRIX-O, although it does not affect the predictive power of the model as a whole. Due to the large sample size, even small associations will become statistically significant which needs to be considered for the interpretation of results as clinically meaningful. The weighting of mental health burden indices may seem somewhat arbitrary, although we applied clinically established cut-offs for psychometric scales. All anonymously made indications elude verification by study design, so we are unable to track hoax responses, although, for instance, only one user reported an age of 99 years.

## Conclusions

Our proof of concept study shows that the risk of job burnout can reliably be assessed with a low-threshold free and anonymous medical health web application. Appropriate means, such as social media entrances, can help targeting users at risk of clinically relevant burnout, thereby offering the possibility of delivering early preventive interventions over the internet. The clinical value of our web application for eHealth purposes awaits evaluation with prospectively collected data, including work- and health related outcomes.

## Abbreviations

BRIX-O: Burnout risk index-original
BRIX-W: Burnout risk index-weighted
ISI: Insomnia severity index
MBI-GS: Maslach burnout inventory- general survey
NA: Negative affect
PA: Positive affect
POMS: Profile of mood states
PSS: Perceived stress scale
